# Chinese Patients’ Intention to Use Different Types of Internet Hospitals: Cross-sectional Study on Virtual Visits

**DOI:** 10.2196/25978

**Published:** 2021-08-13

**Authors:** Liyun Liu, Lizheng Shi

**Affiliations:** 1 School of Humanities and Management Zhejiang Chinese Medical University Hangzhou China; 2 School of Public Health and Tropical Medicine Tulane University New Orleans, LA United States

**Keywords:** internet hospital, direct-to-consumer telemedicine, virtual visit, trust, intention to use, sponsorship type

## Abstract

**Background:**

The issuing of regulation schemes and the expanding health insurance coverage for virtual visits of internet hospitals would incentivize Chinese providers and patients to use virtual visits tremendously. China’s internet hospitals vary in sponsorship. However, little is known about patients’ intention to use virtual visits delivered by different sponsorship types of internet hospitals.

**Objective:**

The goal of the research is to examine patients’ intention to use virtual visits, as well as virtual visits delivered by different sponsorship types of internet hospitals. In addition, we will identify determinants of patients’ intention to use virtual visits, as well as intention to use virtual visits delivered by different sponsorship types of internet hospitals.

**Methods:**

A cross-sectional survey of 1653 participants was conducted in 3-tier hospitals in 3 cities with different income levels in May and June 2019. Binary logistic regression analysis was used to identify the factors that affect patients’ intention to use virtual visits. Multinomial logistic regression analysis was conducted to identify the determinants of the intention to use virtual visits delivered by different sponsorship types of internet hospitals (ie, enterprise-sponsored, hospital-sponsored, and government-sponsored).

**Results:**

A total of 76.64% (1145/1494) of adult participants were online medical information seekers, and 87.06% (969/1113) of online medical information seekers had intention to use virtual visits. Public hospital–sponsored internet hospitals were the most prevalent ones among Chinese patients (473/894, 52.9%), followed by the provincial government internet hospital platform (238/894, 26.6%), digital health companies (116/894, 13.0%), medical e-commerce companies (48/894, 5.4%), private hospitals (13/894, 1.5%), and other companies (6/894, 0.7%). Gender, education, monthly income, and consumer type were significantly associated with the intention to use virtual visits. Gender, age, education, city income level, consumer type, and trust in the sponsor of a health website were significantly associated with the patient’s intention to use virtual visits delivered by 3 different sponsorship types of internet hospitals.

**Conclusions:**

Chinese patients who were online medical information seekers had high intention to use virtual visits and had different intentions to use virtual visits delivered by different sponsorship types of internet hospitals. Public hospitals, the government, and digital health companies were the top 3 sponsorship types of internet hospitals that patients had intention to use. Trust in a health website sponsor significantly influenced the patient’s intention to use virtual visits delivered by different sponsorship types of internet hospitals. Gender, education, and consumer type were the factors significantly associated with both the intention to use virtual visits and the intention to use virtual visits delivered by different sponsorship types of internet hospitals.

## Introduction

### Background

Virtual consultation and virtual visit are two primary types of services delivered by internet hospitals in China. An internet hospital is, to a large extent, an equivalent of direct-to-consumer telemedicine. China’s first internet hospital officially opened in 2014 [1,2]; however, since then the internet hospital industry witnessed an initial development stage with ups and downs. Virtual consultation was the primary service type of internet hospitals from 2014 to 2018. The difference between virtual consultation and virtual visit primarily resulted from the evolution of China’s internet hospital regulation schemes. The State Council of China issued the guideline on Internet Plus Healthcare of 2018 [3], and the National Health Commission accordingly issued specific regulation schemes for online medical diagnosis and treatment as well as internet hospitals in 2018 [4]. The issuing of regulation schemes have brought the rapid development of internet hospitals [5], especially for the service type of online medical diagnosis and treatment. Online medical diagnosis and treatment (hereinafter referred to as virtual visit) is very similar to a virtual visit of direct-to-consumer telemedicine/telehealth in many other countries like the United States because physicians are able to diagnose, treat, and prescribe for some common conditions and chronic diseases for non–first-visit patients. However, physicians are not allowed to diagnose, treat, and prescribe in the virtual consultation service.

In addition to the difference in scope of service, virtual visit is also different from consultation in health insurance coverage. Previous research has suggested that health insurance coverage is a significant factor that affects the use of virtual visits [6]. Following the guideline on Internet Plus Healthcare, in August 2019, the National Healthcare Security Administration of China, as the single payer, has accordingly issued specific guidelines aiming to expand insurance coverage for virtual visit services of internet hospitals [7]. However, consultation services of internet hospitals have not been covered by health insurance. Obviously, health insurance coverage would incentivize both providers and consumers to use virtual visits tremendously. However, there is a lack of studies that specifically examine consumers’ intention to use virtual visits in China.

Internet hospitals vary in sponsors. There are primarily brick-and-mortar hospital-sponsored and enterprise-sponsored internet hospitals in China’s direct-to-consumer telemedicine market [5]. Recently, a very small number of local governments have taken initiatives to set up local government internet hospital platforms by pooling local public health care resources. Hence, enterprises, hospitals and governments are 3 major sponsor types of internet hospitals. Han et al [8] demonstrated that different initiators/sponsors of internet hospitals including the government, hospitals, and enterprises have different purposes and scopes of service (ie, target consumer). The virtual visit market is an emerging market in China, and hence a study on consumers’ intention to use virtual visits delivered by different sponsorship types of internet hospitals is critically important to understand the market structure and future development of the industry. However, little is known about consumers’ intention to use different sponsorship types of internet hospitals in China or intention to use virtual visits delivered by different sponsorship types of internet hospitals.

### Study Aims

We conducted a cross-sectional survey in Zhejiang province to examine patients’ intention to use virtual visits and their intention to use virtual visits delivered by different sponsorship types of internet hospitals and identify the factors that affect patients’ intention to use virtual visits. Zhejiang takes a leading role in China’s internet hospital industry development because it has the first licensed enterprise-sponsored internet hospital (WeDoctor Group) [9] and the first public tertiary hospital–sponsored one (the First Affiliated Hospital of Zhejiang University) [10], the first direct-to-consumer provincial government internet hospital platform [11].

When our survey was conducted, Zhejiang was the first and only province in China that delivered direct-to-consumer telemedicine services including virtual visits for its residents via its provincial internet hospital platform. Only after the outbreak of COVID-19 did other provincial governments start to deliver direct-to-consumer telemedicine service to their residents; however, the majority of services were specially designed free virtual consultations to contain the COVID-19 epidemic. Zhejiang is currently still the leading province to deliver virtual visits by pooling all local health care resources via its provincial internet hospital platform. Therefore, a survey on residents in Zhejiang Province can provide prospective insights for research questions on Chinese consumers’ intention to use virtual visits delivered by hospital-sponsored, enterprise-sponsored, and government-sponsored internet hospitals.

### Literature Review and Hypotheses

As two primary service types of internet hospitals, virtual visit and virtual consultation are different from each other in service scope and health insurance coverage. However, there is hardly a clear division between virtual consultation and virtual visit in the current knowledge of internet hospital literature. Previous studies have examined either patients’ intention to use an internet hospital [12,13] or a virtual consultation delivered by internet hospitals [14]. As noted in the introduction, a virtual visit in China is equivalent to a direct-to-consumer telemedicine/telehealth visit in many other countries. However, most previous studies in the literature have examined patients’ intention to use or actual use of direct-to-consumer telemedicine from the perspectives of specific disease-related application [15,16] and specific telemedicine website/app [17,18]. There is a lack of studies on patients’ decision on sponsorship types of internet hospital platforms and determinants of patients’ decision making.

Systematic reviews have revealed that the technology acceptance model (TAM) was the most commonly used model to examine users’ acceptance of health information technologies including telemedicine [19-21]. The TAM beliefs (technological issues: perceived ease of use, perceived usefulness) and consumer trust are two distinct sets of beliefs that contribute in their own right to increase intention to use the website and, through it, transactions with the e-vendor [22]. Through a systematic review and meta-analysis, Tao et al [19] found that trust is significantly correlated with behavioral intention to use consumer-oriented health information technologies including telemedicine. Trust positively influences the patient’s intention to use telemedicine services in developing countries like Pakistan [23]. However, very few previous studies have examined the patient’s trust in a telemedicine service including trust in the care organization, trust in the care professional, trust in the treatment, and trust in the technology [24]. There is a lack of studies that examine patients’ trust in the sponsor/owner. Based on the abovementioned findings, we hypothesized that trust in the sponsor significantly affected Chinese patients’ intention to use virtual visits delivered by different sponsorship types of internet hospitals (Hypothesis 1).

Websites, apps and WeChat public accounts are 3 primary modalities through which Chinese providers deliver direct-to-consumer online health information and services. Health websites and apps in China usually cover provisions of health/medical information and knowledge, in-person visit online appointment, result tracing of laboratory and diagnostic imaging tests, and consultation, etc. For some health websites that have been licensed to deliver internet hospital services, the internet hospital, especially a virtual visit, is also an essential part of these health websites. However, among all types of online health information services, Chinese patients had the highest awareness and use of in-person visit appointments and medical fee payment online [25]. Therefore, internet hospital service is an important part of health websites, but it is still not an essential part of many health websites in China, especially before the outbreak of COVID-19. Accordingly, most Chinese patients only had prior knowledge or experience of health websites, which led to the result that they formed perceived trust in health websites rather than internet hospital platforms.

The classification of health websites is also different from that of internet hospitals in the current literature. The website owner/sponsor has been identified as one of the most widely reported indicators consumers applied to evaluate the quality of online health information [26,27]. Internet users’ perceived trust in online health information and service delivered by the health website varies by its sponsor/owner type. Health websites vary in their content and features, across commercial, governmental, and nonprofit websites, as they must respond to different structural incentives and constraints, motivations, and purposes [28,29]. Although there are some differences in sponsorship categories of health websites in previous studies, health websites are usually categorized as commercial, governmental, organizational (eg, nonprofit medical institutions), educational (universities and academic institutions), and personal [29-31]. Users usually perceived medical institutions, universities, and governments as the more trustworthy health website sponsors/owners [32]. Based on the findings that users’ trust in health websites varied by the website sponsorship type and trust affected behavioral intention, we concluded that patients’ trust in health website sponsor affected their intention to use health websites and patients’ intention to use a health website varied by its sponsorship type. Based on the fact that an internet hospital was an important part of a health website, we hypothesized that patients’ intention to use virtual visits varied by sponsorship types of internet hospitals (Hypothesis 2).

## Methods

With reference to the Health Information National Trends Survey (HINTS) of the US National Cancer Institute, we made some modifications in accordance with China’s context and research questions to design our survey questionnaire (Multimedia Appendix 1).

### Participants and Data Collection

We conducted a cross-sectional survey using stratified sampling in 3 cities of different income levels in Zhejiang Province, China. According to Zhejiang Statistical Yearbook 2019 [33], 5 cities were categorized as high-income cities (per capita gross domestic product [GDP] > US $15,000), 3 cities were categorized as medium-income cities (US $15,000 ≥ per capita GDP ≥ US $10,000), and 3 cities were categorized as low-income cities (per capita GDP < US $10,000). Proportionate sampling was used to determine the weight of each income-level city stratum. The weight of the stratum was the proportion of the population contained in that stratum, and the population was the urban population of Zhejiang Province in 2018 [33]. The sampling weight of high-income cities was 52.72%, that of medium-income cities was 24.24%, and that of low-income cities was 23.04%.

A pretest with a sample of 39 participants was conducted in April 2019. Based on China’s 3-tier health care delivery system, hospitals are designated as primary, secondary (secondary 2A, 2B), and tertiary institutions (tertiary 3A, 3B). This study conducted an in-person structured questionnaire survey in 5 different types of hospitals (tertiary 3A, 3B, secondary 2A, 2B, and primary) in May and June 2019. A total of 1653 participants were then randomly surveyed in the outpatient center of each hospital, and health practitioners were excluded from the survey. Hangzhou was the high-income city (n=871), Jinhua was the medium-income city (n=401), and Lishui was the low-income level city (n=381).

### Measure

#### Sociodemographic Characteristics

Sociodemographic characteristics of gender, age, education level, and marital status were included in the analysis. Monthly income level (low-income: less than CNY 1800 [less than US $272]; medium-low income: CNY 1801-4600 [US $272-$696]; medium-income: CNY 4601-8000 [US $697-$1210]; medium-high-income: CNY 8001-17,000 [US $1211-$2571]; and high-income: more than CNY 17,000 [more than US $2571]) was included. Participants were asked to rate their current health status using a 5-point Likert scale ranging from very good (1) to very poor (5).

#### Internet Use and Medical Information Seeking

According to Internet Development Report of Zhejiang Province 2019, 80.9% of its residents had access to the internet [34]. Therefore, we modified the HINTS question measuring internet use by asking: “Is the internet your major source of information?” Those participants answering yes were subsequently referred to as active internet users. We modified HINTS questions concerning medical/health information seeking by asking: “Have you used the internet to look for medical information before?” Those who answered yes were subsequently referred to as online medical information seekers; those answered no were excluded from the final analysis.

#### Trust in Health Website Sponsor

We measured the trust in health website sponsorship types among Chinese patients by asking: “Which type of health website do you perceive as the most trustworthy source of medical information?” Websites of medical schools/universities and academic institutions do not provide direct-to-consumer online medical information and services for lay persons; consequently, there are no educational health websites in China. Based on China’s context, we categorized health websites into 4 website sponsorship types: governmental, commercial (digital health companies and internet companies, etc), organizational (hospitals), and personal (individual health practitioners). The option of other was included in case there were some participants who had no prior experience and knowledge of health websites. Rice et al [28] referred to health websites of nonprofit sectors as organizational. In terms of capacities, the majority of hospitals in China are nonprofit public hospitals [35], and there are few other nonprofit organizations providing online direct-to-consumer medical/health information for lay persons. Therefore, the organizational health website thereafter denoted public hospitals.

#### Intention to Use Virtual Visits

We measured the intention to use virtual visits of internet hospitals by asking: “The government has issued guidelines on the development of internet hospitals. Virtual visits of internet hospitals enable physicians to diagnose, treat, and prescribe for some common conditions and chronic diseases for non–first-visits and deliver the prescription right to your door. Do you have the intention to use the virtual visit?”

#### Intention to Use Virtual Visits Delivered by Different Sponsorship Types of Internet Hospitals

We measured the intention to use virtual visits delivered by different sponsorship types of internet hospitals by asking: “Which type of internet hospital do you have the highest intention to use regarding a virtual visit?” There were the facts regarding sponsorship types of internet hospitals in China: (1) hospital sponsors included public hospitals and private hospitals; enterprise sponsors included digital health companies, internet tech companies, medical e-commerce companies, medical informatics companies, health management companies, hospital management companies, pharmaceutical companies, insurance companies, medical equipment companies, etc [5] and (2) the majority of sponsors of enterprise-sponsored internet hospitals were digital health companies and medical e-commerce companies by June 2019 [36]. Therefore, sponsorship types of internet hospitals included 6 options: digital health companies (eg, WeDoctor, Haodf); medical e-commerce companies (eg, Ali Health, JD Health); other companies (enterprise sponsors except digital health companies and medical e-commerce companies); public hospitals; private hospitals; and the Zhejiang Provincial Internet Hospitals Platform.

### Statistical Analyses Strategy

Stata 12.0 software (StataCorp LLC) was used to conduct statistical analyses. Descriptive statistics identified sociodemographic characteristics of the final sample, proportions of participants who had the intention to use virtual visits, and proportions of participants who had the intention to use virtual visits delivered by different sponsorship types of internet hospitals.

A systematic review of end user acceptance of telemedicine use [21] found that logistic regression, structural equation modeling, and linear regression were 3 primary statistical analysis methods conducted in previous studies. It also found that TAM was the most used model. Structural equation modeling was primarily used to examine the relationships between factors like TAM beliefs (perceived ease of use, perceived usefulness) and behavioral intention to use telemedicine. And there were usually several paths through which different factors affected the behavioral intention. However, in this study, there was only one path (ie, the trust in sponsor affected patients’ intention to use virtual visits delivered by different types of internet hospitals). Additionally, the sponsorship type of internet hospital was a qualitative variable (categorical variable). Therefore, logistic regression was the appropriate statistical technique to identify the determinants. Binary logistic regression analysis was used to identify the factors that affect patients’ intention to use the virtual visit. Multinomial logistic regression analysis was conducted to examine the association of patient characteristics with the intention to use virtual visits delivered by different sponsorship types of internet hospitals.

## Results

### Sample Selection

**Figure 1:**
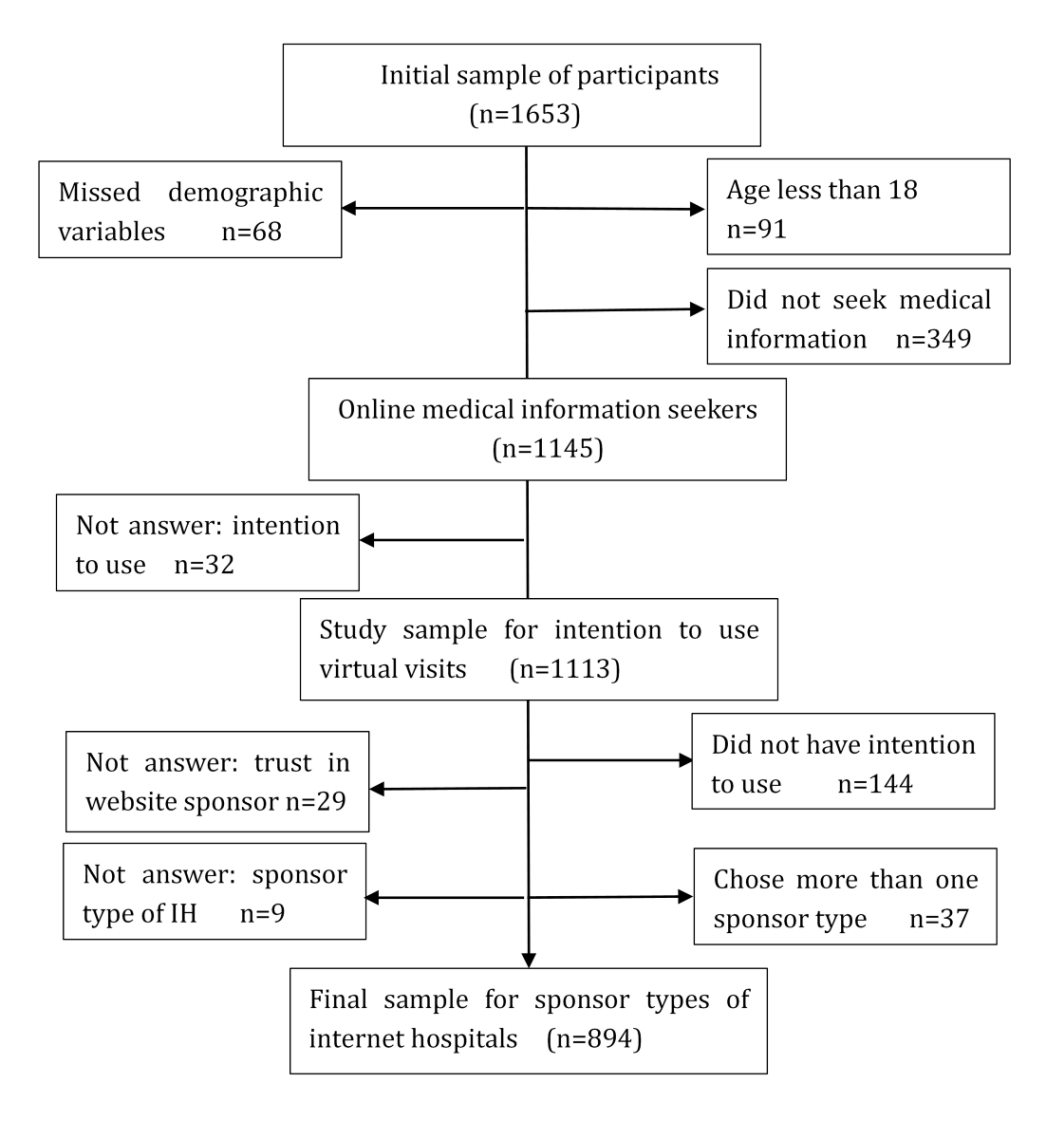
Flowchart of sample selection. IH: internet hospital.

Health information seeking was the patient’s most frequently used eHealth or mobile health (mHealth) activity [37], and health information seeking was also a significant predictor of eHealth and mHealth service use [38,39], therefore we inferred that online medical information seekers were target consumers for internet hospitals. To identify the target market, 349 participants were excluded because they self-reported that they have not sought medical information online before. The study sample for statistical analysis on intention to use virtual visits was 1113. The final sample for statistical analysis on intention to use virtual visits delivered by different sponsorship types of internet hospitals was 894. Figure 1 shows the flowchart of sample selection.

### Sociodemographic Characteristics

Among adult participants, 76.64% (1145/1494) were online medical information seekers. Among medical information seekers, those who did not answer the question of intention to use were excluded. Table 1 shows the sociodemographic characteristics of the final sample for intention to use virtual visits. There were more female medical information seekers than male seekers, which was possibly because females were more likely to be caregivers and information seekers who accompanied family members to visit hospitals. The majority (767/1113, 68.91%) of seekers had the education level of college graduate. Young seekers aged 18 to 55 years accounted for 98.11% (1092/1113) of participants. The survey was conducted in cities in Zhejiang Province where cities usually had both high stocks and inflows of well-educated young people. The majority (965/1113, 86.71%) of seekers were in good or fair health status. Compared to other variables, monthly income was more equally distributed. A total of 80.41% (895/1113) were active internet users whose major information source was the internet. A total of 87.06% (969/1113) reported that they had intention to use virtual visits of internet hospitals.

**Table 1:** The sociodemographic characteristics of study sample for intention to use virtual visits (n=1113).

Characteristic		Value, n (%)
**Gender**		
	Male	445 (39.98)
	Female	668 (60.02)
**Age (years)**		
	18-29	508 (45.64)
	30-40	430 (38.63)
	41-55	154 (13.84)
	56-65	15 (1.35)
	>65	6 (0.54)
**Health status**		
	Very good	110 (9.88)
	Good	482 (43.31)
	Fair	483 (43.40)
	Poor	36 (3.23)
	Very poor	2 (0.18)
**Marital status**		
	Married	658 (59.12)
	Single	434 (38.99)
	Divorced	19 (1.71)
	Widowed	2 (0.18)
**Education level**		
	≤Junior high school	101 (9.07)
	Senior high school	166 (14.91)
	College graduate	767 (68.91)
	Postgraduate	79 (7.11)
**Monthly income (CNY)**		
	≤1800	122 (10.96)
	1801-4600	326 (29.29)
	4601-8000	350 (31.45)
	8001-17,000	226 (20.30)
	>17,000	89 (8.00)
**Internet use**		
	Inactive user	218 (19.59)
	Active user	895 (80.41)
**Intention to use virtual visits**		
	No	144 (12.94)
	Yes	969 (87.06)

### Intention to Use Virtual Visits

The binary logistic regression result of intention to use virtual visits of internet hospitals is presented in Table 2. The female was more likely to have intention to use virtual visits (odds ratio [OR] 1.68, 95% CI 1.13-2.48). Education level was significantly and positively associated with the intention to use virtual visits, senior high school (OR 2.05, 95% CI 1.09-3.86), college graduate (OR 3.44, 95% CI 1.94-6.12), postgraduate (OR 3.09, 95% CI 1.19-8.00). Patients with high income level were more likely than those with low income level to have intention to use virtual visits (OR 3.22, 95% CI 1.13-9.15). Consumers of tertiary B hospitals were more likely than consumers of primary hospitals to have intention to use virtual visits (OR 2.62, 95% CI 1.12-6.13).

**Table 2:** Binary logistic regression result of intention to use virtual visits of internet hospitals (n=1113).

Characteristic		OR^a^ (95% CI)	*P* value
Gender (ref^b^: Male)		1.68 (1.13-2.48)	.01
**Age (ref: 18-29)**			
	30-40	0.92 (0.54-1.58)	.77
	41-55	1.18 (0.58-2.38)	.65
	56-65	0.62 (0.15-2.53)	.51
	>65	0.71 (0.11-4.65)	.72
Health status		1.14 (0.89-1.47)	.29
**Marital status (ref: Married)**			
	Single	0.75 (0.44-1.30)	.31
	Divorced	0.69 (0.20-2.33)	.55
	Widowed	^—c^	—
**Education level (ref: ≤Junior high school)**			
	Senior high school	2.05 (1.09-3.86)	.03
	College graduate	3.44 (1.94-6.12)	<.001
	Postgraduate	3.09 (1.19-8.00)	.02
**Monthly income (CNY; ref: ≤1800)**			
	1801-4600	1.00 (0.54-1.83)	.99
	4601-8000	1.67 (0.88-3.17)	.12
	8001-17,000	1.51 (0.75-3.03)	.25
	>17,000	3.22 (1.13-9.15)	.03
Internet use (ref: Inactive)		1.37 (0.88-2.13)	.17
**City (ref: Hangzhou)**			
	Jinhua	1.37 (0.80-2.35)	.25
	Lishui	1.26 (0.73-2.18)	.41
**Hospital type (ref: Primary)**			
	Secondary B	0.64 (0.23-1.82)	.41
	Secondary A	1.89 (0.71-5.09)	.21
	Tertiary B	2.62 (1.12-6.13)	.03
	Tertiary A	1.95 (0.94-4.06)	.07
Constant		0.45 (0.13-1.54)	.20

^a^OR: odds ratio.

^b^ref: reference group.

^c^Not applicable.

### Intention to Use Virtual Visits Delivered by Different Types of Internet Hospitals

Table 3 presents Chinese patients’ intention to use virtual visits delivered by different types of internet hospitals. Public hospital–sponsored internet hospitals were the most prevalent (473/894, 52.9%), followed by the provincial government internet hospital platform (238/894, 26.6%), digital health companies (116/894, 13.0%), medical e-commerce companies (48/894, 5.4%), private hospitals (13/894, 1.5%), and other companies (6/894, 0.7%). This result was consistent with the current market structure of internet hospitals in China. Brick-and-mortar hospital sponsors and enterprise sponsors were 2 primary sponsor types of internet hospitals in China, while hospital-sponsored ones accounted for 83.5% (415/497) of internet hospitals by April 30, 2020. Within the category of hospital-sponsored internet hospitals, public hospitals and private hospitals accounted for 90.4% (375/415) and 9.6% (40/415) [5].

Multinomial logistic regression was used to identify the determinants of intention to use virtual visits delivered by 3 major different sponsorship types of internet hospitals (enterprise-sponsored, hospital-sponsored, and government-sponsored). The final sample for the multinomial logistic regression analysis was 894. Table 4 shows the result of multinomial logistic regression analysis.

Using hospital-sponsored internet hospitals as the base outcome, we had following results. Females were more likely to have intention to use virtual visits delivered by the governmental internet hospital platform and less likely to have intention to use visits delivered by enterprise-sponsored internet hospitals. Participants aged 30 to 40 years were more likely than those aged 18 to 29 years to have intention to use the governmental internet hospital platform. Participants with higher education levels were less likely to have intention to use virtual visits delivered by enterprise-sponsored internet hospitals. Participants in a medium-income city (Jinhua) were less likely than those in a high-income city (Hangzhou) to have intention to use virtual visits delivered by both enterprise-sponsored internet hospitals and governmental internet hospital platform. Consumers of secondary A, tertiary B, and tertiary A hospitals were less likely than consumers of primary hospitals to have intention to use enterprise-sponsored internet hospitals. Participants who trusted commercial health websites most were more likely than those trusted governmental health websites to have intention to use virtual visits delivered by enterprise-sponsored internet hospitals rather than visits delivered by hospital-sponsored ones. Compared to participants who trusted in governmental health websites, participants who trusted all other sponsorship types of health websites were less likely to use virtual visits delivered by the governmental internet hospital platform.

**Table 3:** Patients’ intention to use virtual visits delivered by different types of internet hospitals (n=894).

Internet hospital type		Value, n (%)
**Enterprise-sponsored**		170 (19.0)
	Digital health companies	116 (13.0)
	Medical e-commerce companies	48 (5.4)
	Other companies^a^	6 (0.7)
**Hospital-sponsored**		486 (54.4)
	Public hospitals	473 (52.9)
	Private hospitals	13 (1.5)
Government-sponsored		238 (26.6)

^a^Other companies: all other enterprise sponsors except digital health companies and medical e-commerce companies.

**Table 4:** Multinomial logistic regression result of intention to use virtual visits delivered by different sponsorship types of internet hospitals (base outcome=hospital; n=894).

Characteristic			Enterprise				Government		
			RRR^a^ (95% CI)	*P* value		RRR (95% CI)			*P* value
Gender (ref^b^: Male)			0.66 (0.44-0.98)	.04		1.57 (1.09-2.28)			.02
**Age (years; ref: 18-29)**									
	30-40	1.01 (0.59-1.74)		.96	1.74 (1.07-2.83)			.03	
	41-55	0.75 (0.36-1.56)		.44	0.83 (0.42-1.63)			.59	
	56-65	1.85 (0.36-9.36)		.46	0.54 (0.05-6.19)			.62	
	>65	1.39 (0.10-18.34)		.80	—^c^			.99	
Health status			0.86 (0.66-1.12)	.25		1.04 (0.82-1.32)			.73
**Marital status (ref: Married)**									
	Single	1.36 (0.80-2.33)		.25	1.40 (0.86-2.27)			.18	
	Divorced	0.50 (0.06-4.25)		.52	1.19 (0.36-3.91)			.78	
	Widowed	—		.99	—			.99	
**Education (ref: ≤Junior high school)**									
	Senior high school	0.33 (0.14-0.77)		.01	0.67 (0.29-1.53)			.34	
	College graduate	0.46 (0.22-0.96)		.04	0.97 (0.45-2.09)			.94	
	Postgraduate	0.20 (0.06-0.61)		.01	0.73 (0.27-1.98)			.54	
**Monthly income (CNY; ref: ≤1800)**									
	1801-4600	0.83 (0.42-1.63)		.58	0.72 (0.39-1.32)			.29	
	4601-8000	0.63 (0.31-1.26)		.19	0.87 (0.48-1.60)			.66	
	8001-17,000	1.58 (0.76-3.31)		.22	1.25 (0.64-2.45)			.51	
	>17,000	0.73 (0.28-1.89)		.52	0.66 (0.29-1.50)			.32	
Internet (ref: Inactive)			1.10 (0.65-1.86)	.73		0.76 (0.49-1.17)			.22
**Trust in health website sponsor (ref: Governmental)**									
	Organizational	0.79 (0.44-1.44)		.44	0.41 (0.27-0.62)			<.001	
	Commercial	3.61 (1.87-6.97)		<.001	0.28 (0.15-0.54)			<.001	
	Personal	0.54 (0.16-1.76)		.30	0.37 (0.16-0.89)			.03	
	Other	1.51 (0.63-3.61)		.35	0.32 (0.14-0.74)			.01	
**City (ref: Hangzhou)**									
	Jinhua	0.53 (0.31-0.91)		.02	0.58 (0.36-0.92)			.02	
	Lishui	0.88 (0.50-1.55)		.66	0.72 (0.45-1.15)			.17	
**Hospital type (ref: Primary)**									
	Secondary B	0.68 (0.20-2.30)		.54	1.64 (0.47-5.66)			.44	
	Secondary A	0.27 (0.10-0.74)		.01	1.24 (0.43-3.53)			.69	
	Tertiary B	0.46 (0.19-1.10)		.08	1.69 (0.67-4.26)			.27	
	Tertiary A	0.33 (0.15-0.73)		.01	1.51 (0.64-3.54)			.34	
Constant			3.52 (0.78-15.91)	.10		0.64 (0.15-2.78)			.55

^a^RRR: relative risk ratio.

^b^ref: reference group.

^c^ Not applicable.

## Discussion

### Principal Findings

Chinese patients who were online medical information seekers had a high intention to use virtual visits of internet hospitals. Gender, education, monthly income, and consumer type were significantly associated with the intention to use virtual visits. Patients had different intentions to use virtual visits delivered by different sponsorship types of internet hospitals, in which the public hospital–sponsored one was the most prevalent one, followed by the government, digital health companies, medical e-commerce companies, private hospitals, and other companies. Gender, age, education, city income level, consumer type, and trust in health website sponsor were significantly associated with the patient’s intention to use virtual visits delivered by 3 different sponsorship types of internet hospitals (enterprise-sponsored, hospital-sponsored, and government-sponsored).

### Comparison With Prior Work

#### Intention to Use Virtual Visits

This study, to our knowledge, was the first survey that examined the intention to use virtual visits delivered by different sponsorship types of internet hospitals among patients in China. Previous studies have examined the intention to use internet hospitals in China; however, most of them have not specifically examined the intention to use virtual visits of internet hospitals [12,13]. This study revealed that 87.06% of patients who were online medical information seekers had intention to use virtual visits of internet hospitals, which was much higher than the finding by Li et al [12] that showed 65.6% of participants were willing to use internet hospitals. Our statistical analysis examined online medical information seekers’ willingness to use virtual visits; therefore, it was very likely that the intention to use was much higher than the study by Li et al [12]. This large difference could be explained partly by the place where the survey has been conducted, since our survey was conducted in Zhejiang Province, while the survey by Li et al [12] was conducted in Sichuan Province, whose internet development index lagged far behind Zhejiang Province [40]. This large difference could also be possibly explained by the measurement of intention to use because we pointed out that the government has issued regulation guidelines for internet hospitals, especially virtual visits, in our question to measure the intention to use virtual visits.

#### Intention to Use Virtual Visits Delivered by Different Sponsorship Types of Internet Hospitals

Findings of this study have confirmed Hypothesis 2 that patients had different intentions to use virtual visits delivered by different sponsorship types of internet hospitals, as well as Hypothesis 1 that trust in sponsor was the significant determinant of the patient’s intention to use virtual visits delivered by different sponsorship types of internet hospitals. Public hospitals and the government were the top 2 sponsors of internet hospitals in which patients had high intention to use virtual visits, which was consistent with previous studies that found patients tended to trust health websites sponsored by hospitals, universities, government agencies, and well-known nonprofit organizations [32,41]. Enterprise-sponsored internet hospitals lagged behind public hospital–sponsored and government-sponsored ones, which was also consistent with prior studies that found that patients were likely to distrust health websites that appeared to be commercial [42-44]. These findings indicated that TAM was the robust model to explain the patient’s acceptance of different sponsorship types of internet hospitals.

Three different categories of internet hospitals (enterprise-sponsored, hospital-sponsored, and government-sponsored) varied in target customer, motivation, and online health care resource allocation. The government-sponsored internet hospital aims to deliver internet hospital services to all its local residents by pooling almost all its regional public medical institutions. Hospital-sponsored internet hospitals usually confine the health care resource to the individual hospital itself or the medical alliance brought by the integrated delivery system which often includes public hospitals and primary health care institutions [1]. Enterprise-sponsored internet hospitals aim to deliver internet hospital services to consumers nationwide by attracting licensed physicians nationwide, especially from public tertiary hospitals. As noted, despite the difference in target consumer, motivation, and online health care resource allocation, most internet hospitals recruited most of their physicians from the public health care system. There are definitely competitions between different categories, as well as competitions within categories.

Trust (also often referred to as credibility) has 2 primary components: trustworthiness (perceived motivation) and expertise (perceived ability) to provide accurate and truthful information [45]. Patients had the highest intention to use virtual visits delivered by public hospital–sponsored internet hospitals (52.91%) but very low intention for private hospital–sponsored ones (1.45%). This finding was in accordance with the fact that private hospital–sponsored internet hospitals only accounted for 10% of hospital-sponsored internet hospitals [5]. Because public and private hospitals varied in motivations, patients usually perceived private hospitals as less trustworthy, despite the same expertise level. Furthermore, private hospitals in China lacked the accumulation of reputation, patients had impressions that private hospitals were more concerned about economic benefits rather than patients’ benefits, and they fulfilled less social responsibility than public hospitals; tendentious news reports also exacerbated these impressions [46].

Patients had higher intention to use virtual visits delivered by digital health company–sponsored internet hospitals (12.98%) than medical e-commerce company-sponsored ones (5.37%) and other companies (0.67%), which was in accordance with the fact that digital health companies played a dominant place in the enterprise-sponsored internet hospitals (44%) [5]. As noted, digital health companies had particular advantage over all any other companies on the perceived expertise dimension of trust, and thus patients were more likely to trust digital health companies than all other companies, despite the same trustworthiness level. All other companies recently have made efforts to increase their roles in the internet hospital market, especially after the outbreak of COVID-19. Internet tech companies and medical e-commerce companies possessed their critical advantages on the larger user base of their parent firms and easy access to internet end users; we need to wait to see the results of competition within the category of enterprise-sponsored internet hospitals.

#### Determinants of Intention to Use Virtual Visits Delivered by Different Sponsorship Types of Internet Hospitals

The findings on the association of sociodemographic variables and the intention to use telemedicine remained mixed. This study identified that gender, education, income, and consumer type were the factors significantly associated with the intention to use virtual visits. Most previous studies also found that patients who were more willing to use telemedicine tended to have a higher education level [12,47,48]. The majority of prior studies demonstrated that gender had no significant effect on the intention to use telemedicine [12,48]. This study found that females were more likely to have intention to use virtual visits; one possible explanation was that females were more likely to be the family caregiver [49]. Compared to low-income patients and primary hospitals consumers, high-income patients and tertiary hospitals consumers were more likely to have intention to use virtual visits because they had higher demand for high quality of care and internet hospitals provided them an alternative access to high quality care virtually.

This study indicated that gender, education, and consumer type were the factors significantly associated with both the intention to use virtual visits and the intention to use virtual visits delivered by different sponsorship types of internet hospitals. Patients with the characteristics of being female, higher education level, tertiary A and B hospital, and secondary A hospital consumers were more likely to have intention to use virtual visits delivered by hospital-sponsored internet hospitals other than enterprise-sponsored ones. These groups of patients had higher demand for high-quality care in which hospital-sponsored internet hospitals were perceived as a better deliverer than enterprise-sponsored ones.

Females were more likely than males to prefer the government-sponsored internet hospitals to hospital-sponsored ones, as on one hand the government-sponsored one was more convenient for females (the primary family caregiver) because it pooled the regional public hospitals together; on the other hand, female Chinese were more likely to trust in governments [50]. Patients aged 30 to 40 years were more likely than those aged 18 to 29 years to have intention to use the government-sponsored internet hospital; one possible explanation was that they attached more importance to convenience.

Compared to enterprise-sponsored and government-sponsored internet hospitals, patients in medium-income cities were more likely than those in high-income cities to have intention to use virtual visits delivered by hospital-sponsored ones. Zhejiang Province has done a good job in market penetration, since there are quite a few internet hospitals at municipal level and even at county level [5]. The more developed the city is, more tertiary hospitals the city has. However, virtual visits only cover some common conditions and chronic diseases for non–first-visits; the demand of the patient in a medium-income city would be met by the city’s public tertiary hospitals, many of which have set up internet hospitals.

### Limitations and Future Research

This study has several limitations. First, this study specifically examined online medical information seekers’ intention to use virtual visits delivered by internet hospitals, which might overestimate the whole group of patients’ intentions to use. Second, there are urban-rural divides at various aspects in China. The survey was conducted in cities of Zhejiang Province. As rural population was not included in this survey, the findings of this study only revealed urban patients’ behavioral intention toward virtual visits. We will extend our study by conducting the survey on the whole population (both urban and rural population, both online and nononline medical information seekers) in the future.

Third, Zhejiang provincial internet hospital platform was initially launched on Alipay (the digital payment platform of Alibaba Group) in January 2019. Our survey was conducted in May and June 2019, and in July 2019, the Zhejiang provincial internet hospital platform was transferred to the all-in-one digital provincial government website/app. This transfer might have some effects on patients’ intention to use the government-sponsored internet hospital platform.

Fourth, this study was conducted before the outbreak of COVID-19, which may have been greatly different from the current situation. The outbreak of COVID-19 has brought tremendous growth of internet hospitals in China and has drastically increased the awareness of virtual visits. The rapid increase of awareness would greatly increase the patient’s intention to use and actual use of virtual visits. We will follow and further the study by continuous survey on patients’ behavioral beliefs, intention to use, and actual use of internet hospitals to measure the impacts of drastic expansion of different sponsorship types of internet hospitals (especially public hospital–sponsored ones) brought by the outbreak of COVID-19.

### Conclusion

This study has demonstrated that virtual visits differ from virtual consultations in both service scope and health insurance coverage. The virtual visit market is an emerging market in China. This study has implied that different sponsorship types of internet hospitals (ie, enterprises, hospitals, and the government) have different motivations, target consumers, and online health care resource allocation. This study found that Chinese patients who were online medical information seekers had high intention to use virtual visits. Public hospitals, the government, and digital health enterprises were the top 3 sponsorship types of internet hospitals in which patients had intention to use their virtual visit services.

This study revealed that trust in a health website sponsor significantly influenced the patient’s intention to use virtual visits delivered by different types of internet hospitals, which extended the current knowledge regarding the impact of trust on adoption of direct-to-consumer telemedicine service. This study implied that internet hospitals should pay more attention to consumers with characteristics of being female and a tertiary hospital consumer with higher education level and high income level when developing the virtual visit market. It also implied that different sponsorship types of internet hospitals had different target consumers for virtual visit service. Hospital-sponsored internet hospitals had an advantage over the government-sponsored and enterprise-sponsored ones when developing the virtual visit market in medium-income cities.

## Appendix

Multimedia Appendix 1 Questionnaire (in Chinese).

## Abbreviations

GDP: gross domestic product
HINTS: Health Information National Trends Survey
mHealth: mobile health
OR: odds ratio
TAM: technology acceptance model
