# Supplementary material for: Chinese Patients’ Intention to Use Different Types of Internet Hospitals: Cross-sectional Study on Virtual Visits
Source: J Med Internet Res. 2021 Aug 13;23(8):e25978. doi: 10.2196/25978 (PMC8398707; doi:10.2196/25978)
Supplement: Multimedia Appendix 1 [file jmir_v23i8e25978_app1.doc]

**浙江省居民互联网医院使用意愿调查**

**您好！**

我们是浙江中医药大学的学生，为了优化医疗信息服务平台，现开展一项关于浙江省居民互联网医疗信息和服务需求的调查，以了解浙江省居民互联网医疗信息和服务的多元需求。本问卷不涉及调查对象隐私，结果仅用于学术研究，请您放心填写。请您在选择的答案前打勾。谢谢!

1 您的性别是

A、男 B、女

2 您的年龄是

A、18岁以下 B、18岁到29岁 C、30岁到40岁

D、41岁到55岁 E、56岁到65岁 F、65岁以上

3 您觉得自己的健康状况怎么样？

A、很好 B、好 C、一般 D、不好 E、很不好

4 您的婚姻状况是？

A、已婚 B、未婚 C、离异 D、丧偶

5 您的受教育程度是？

A、小学及小学以下 B、初中 C、高中

D、大学本科/专科 E、研究生及以上

6 您的月收入大概是多少？

A、1800元以下 B、1801元—4600元 C、4601元—8000元

D、8001元—17000元 E、17000元以上

7 互联网是您主要的信息来源吗？

A、是 B、不是

8 您有没有上网查询过医疗信息？

A、有 B、没有 **（选没有，问卷结束，谢谢！）**

9 下列医疗健康信息平台提供的信息，您**最信任**哪一种？**单选**

A、互联网医疗企业等企业的信息平台（如平安好医生、好大夫等）

B、医院官方网站或者平台

C、卫生部门官方网站或平台

D、医生个人的网页、微博或者微信公众号

E、其他

10 政府推动实体医疗机构开设互联网医院，开展部分常见病、慢性病复诊等互联网诊疗服务（网上复诊、开电子处方、送药上门），您愿意接受吗？

A、愿意 B、不愿意**（问卷结束，谢谢！）**

11 您最愿意选择下面哪一种类型的互联网医院的在线诊疗服务？**单选**

A、好大夫、微医等互联网医疗企业

B、阿里健康、京东健康等医药电商

C、浙一医院、金华市及丽水市人民医院等公立实体医院

D、民营实体医院

E、浙江省互联网医院平台（卫生部门授权、支付宝入口、将统一浙江全省实体医疗机构的互联网医院服务）

F、其他（企业的平台）

非常感谢您对本次调查的支持！
